# How stakeholder participation can contribute to systematic reviews of complex interventions

**DOI:** 10.1136/jech-2015-205701

**Published:** 2015-10-16

**Authors:** J Harris, L Croot, J Thompson, J Springett

**Affiliations:** 1 University of Sheffield, School of Health & Related Research, Sheffield, England; 2 University of Sheffield, School of Nursing & Midwifery, Sheffield, England; 3 University of Alberta, Centre for Health Promotion Studies, School of Public Health, Alberta, Canada

**Keywords:** PUBLIC HEALTH, RESEARCH METHODS, HEALTH PROMOTION, SOCIAL INEQUALITIES

## Abstract

Although patient and public involvement in research is a requirement for research funding in many countries, the knowledge base for how to effectively involve people—and evidence of the effectiveness of involvement—is weak. This article describes how methods used in participatory health research were used to involve patients, clients, providers and community health workers across all stages of a realist review. Sustained involvement enabled better identification of the components of the complex intervention of community-based peer support. It also challenged assumptions of how peer support is constructed, leading the review team to question whether the process of designing and implementing interventions has more influence on effectiveness than previously recognised in empirical studies. We conclude with a discussion on when sustained involvement should be used, and the challenges of incorporating it into the traditional researcher-led approach to systematic reviews.

## Introduction

Systematic reviews of health effectiveness have traditionally been undertaken by teams of topic experts, primarily clinicians or health researchers. Nearly 10 years ago, the involvement of service users and carers was referred to as a phenomenon ‘emerging out of the shadows’^1^ which was relatively underdeveloped. Recent research confirms that user involvement remains relatively rare, with few organisations engaging consumers.^2^

In the field of community development, patient and public involvement (PPI) is not new. In the UK, for example, national legislation has reflected an increasing policy drive to involve patients, service users and carers in health research.^3^ ^4^ PPI is regarded as an umbrella term, used to refer to a variety of stakeholders or end users/recipients/beneficiaries of research and means working with patients and the public as members of the research team.

Three rationales for PPI in designing primary health research have been put forward in the literature. First, PPI is suggested as a mechanism to address a perceived democratic deficit in public policy making and research. Second, PPI is suggested as a way to improve public trust in research, ensuring greater transparency and accountability.^5^ Third, the literature suggests that PPI can ensure that research is designed to meet end users’ needs.^6^ It is argued that the public brings a unique contribution to research in terms of their personal knowledge of a particular illness or condition and/or their experiences of services, labelled as their ‘experiential expertise’.^7^ ^8^ Experiential expertise may provide alternative perspectives to those of health professionals and researchers, bringing an element of ‘reality check’ to research. Of course tensions may exist in terms of whose expertise is given more credence. The attitude of the certified expert towards the experiential expert, and vice versa, will often determine whether or not each member is viewed as a credible participant in the research process. Within health and social care research, some researchers are still driven by the epistemology of logical positivism, striving for objectivity and rational claims to universality.^7^ These values can make it difficult for some researchers to view experiential expertise as valid, and involvement can challenge the evidence produced by strict objective methodologies.^9^

Engagement usually occurs when setting research agendas, enrolling participants, and disseminating findings.^5^ Participation in the actual design, delivery and analysis of interventions is far less common. Further, there is only weak evidence indicating that involvement can improve potential to increase relevance, accessibility, accountability, and possibly acceptability of the results.^10^ Evaluation tools that enable involvement to be assessed at all stages of the process are needed. Examples can be drawn from the field of community-based participatory research where a long tradition of community involvement in research is generating a portfolio of tools for assessing impact.^11^

If lack of involvement compromises the effectiveness of single interventions, then systematic reviews may produce equivocal findings because involvement is a ‘hidden ingredient’ impacting on effectiveness. The potential importance of PPI is now being noted in methodological articles discussing systematic reviews of complex interventions. Anderson *et al*^12^ note that reviewers need to know when to involve potential users of the review, the purpose of the involvement, and how to involve them. Methodological experts in systematic reviewing have recently proposed that stakeholders could contribute to reviewing complex interventions at various stages.^13^ Possible ways to be involved include obtaining patient views on important proximal and distal outcomes; developing an understanding of how different components in an intervention interact or connect with one another to produce a synergistic effect; explaining how behaviour change is supported by relationships and communication; understanding how context interacts with the development of capabilities; and clarifying whether review findings can be applied to different populations and settings.^14^ Further, PPI could increase understanding of issues that surround and modify effectiveness, such as acceptability, equity and efficiency.^12^

An emerging methodology for reviewing complex interventions and developing explanations for what works, for whom and in what circumstances is realist review.^15^ The basis of realist review is scientific realism, which proposes that while reality exists independent of the researcher, the knowledge generated is relative to the researcher and cannot be extricated from the surrounding social context.^16^ The approach focuses on analysing the interactions between actors, structures and institutions, in order to identify the mechanisms which enable a successful intervention. It would seem that PPI is uniquely positioned to describe the mechanisms, which are the “underlying entities, processes, or (social) structures which operate in particular contexts to generate outcomes of interest”.^17^

Interestingly, the role of stakeholders, the public or service users is not defined in realist review reporting standards.^18^ Most realist review teams consist of academics, who involve expert practitioners,^19–21^ knowledge users or policymakers^22–24^ at the beginning and end of the process. Both effectiveness reviews and realist reviews appear to frame involvement as a consultant role, with occasional contact. There are less than a handful of realist reviews that are planning involvement or have actively involved patients and service users on a more continuous basis via expert patient groups and advisory panels.^25^ ^26^

The lack of involvement in reviews of complex interventions may be due to the fact that reviewers do not have the skills or time to coordinate meaningful involvement. In Brett *et al*'s^10^ review of the impact of PPI on health research, 14 of the 66 included studies cited time and cost as concerns that added to researchers’ workloads. It therefore seems logical to ask whether certain topics produce more added value than others before including involvement in reviews of complex interventions.

In this article we use a recently funded review of community-based peer support^27^ to ask: how can stakeholders be meaningfully involved in reviews of complex interventions? Is there added value in continuous involvement throughout the review process? We provide an overview of how we used the principles of community based participation in health to construct an Advisory Network that played an essential role in identifying the components of complex interventions and developing theory for community-based peer-support interventions. The experience has led us to propose that for some topics and situations a greater degree of participation is needed in order to produce a contextually valid synthesis.

### Why is additional stakeholder involvement needed in reviews of complex interventions?

Community-based peer support is a complex intervention that achieves effects via the active input of the individuals involved. In much empirical literature human involvement is seen as a contaminant and safeguards such as randomisation, placebos and blinding are used to eliminate the impact of human involvement. In contrast active programmes only work through stakeholder reasoning and personal choices and knowledge of that reasoning is integral to understanding the successes and failures of these interventions. While published empirical research studies tend to focus on tangible processes and formally measurable outcomes, there is a danger that informal information, relating to interpersonal relationships and the subtle contextual conditions which make interventions sink or swim, might be missed. Our review was designed to be a participatory realist review in which primary data were collected from lay experts working in this field, as well as beneficiaries of the intervention and professional health workers. We decided to refer to this as ‘participatory realist synthesis’, because participatory approaches to research allow for prolonged engagement with people who have relevant expertise. We created an Advisory Network spanning providers, commissioners, patients, clients and volunteer workers who contributed a situational understanding of how peer support promotes health literacy. The members provided explanatory detail to complimenting the often sparse reporting in primary research articles. In the next section, we describe the review methods in order to illustrate how participation facilitated comparison of empirically supported and culturally supported interventions.

## Methods for participatory review

### Focusing the review

The funders for our review (the National Institute of Health Research) were interested in exploring the evidence base for community engagement, including the potential for engagement to reduce health inequalities. We selected a common approach to engagement—peer support—and conducted a scoping search to get a better idea of the amount and type of available research. The academic team conducted this search before stakeholder involvement, in order to help us to make decisions about the types of stakeholders who may be able to contribute to the review.

We carried out scoping searches across Scopus, Global Health (including Medline), ProQuest (including ERIC and Social Work Abstracts), King’s Fund Database, Web of Knowledge, and the Institute of Development Studies. The period covered was 1975–October 2011 with language of publication restricted to English only. We did not restrict by country, but were particularly interested in mapping the topics where peer support was used in the UK, in order to inform national community engagement policy. The scoping search returned 570 included papers of which 39 were directly attributable to a UK context and 122 papers related to models and theories. From the scoping search seven UK based topics were identified where peer support is commonly used in interventions with vulnerable groups. The topics included breast feeding, diabetes, older people and healthy living, HIV prevention, nutrition, and smoking cessation. Articles were selected in the first instance for relevance, that is, if they focused on research, evaluation or models of peer-support programmes situated in communities and focusing on improving health. Multiple study designs were needed to illuminate the bigger picture, so for this review no single study design was considered to be dominant over any other, rather the value of each study was judged against criteria relating to explanatory depth and contextual relevance. Within each topic, we identified related articles that had been published describing the same project or programme along with the cited programme theories. This enabled us to compensate somewhat for thin reporting in single publications, producing more conceptual richness. We referred to these bodies of literature as topic ‘clusters’.^28^

### The process of engaging participants

Once we had established common health topics where peer support was used, we established an Advisory Network made up of individuals and organisations known to provide grass roots peer support in each of these health topics. From a participation perspective, it could be argued that stakeholders should be involved from the inception of a review, to focus the search and ensure that research relevant to policy concerns was identified. Our reason for starting the scoping first was entirely pragmatic—it took time to identify peer-support workers. Members of the project team started with existing contacts in the community to identify and approach individuals and organisations, augmenting the process by funding a third sector umbrella Consortium, made up of organisations providing health and well-being services to local people. The Consortium assisted in identifying and recruiting appropriate groups and individuals working in each topic area.

Recruitment took place throughout the review, with some participants contributing on multiple occasions and others making a single contribution at a particular stage. Salaried workers, health trainers, volunteer health champions, and programme coordinators with expertise in using peer support participated, as well as people who had originally received support before going on to become a peer-support worker. Within the Advisory Network there were many aims, purposes and motivations for different peer-support interventions giving us access to a wide range of expertise in providing community-based peer support to promote health and well-being and reduce health inequalities.

### Defining complexity during the review process

We collected descriptions of peer-support interventions via five cross-organisation events as well as seven within-organisation events for groups and individuals who might be disadvantaged or under-represented if asked to attend a mixed group in an unfamiliar setting. In total we made approximately 240 face-to-face contacts with around 120 participants in Yorkshire, the East Midland and London regions. In addition to the events, participants were able to contribute by responding to email discussions or through opportunistic contacts with members of the team.

Questions we set out to answer included:

- What is a peer?
- What makes you a peer?
- What is important in being a peer?
- What components make up peer support?
- What do you do and how do you do it?
- When do you do it and why?

We used participatory methods and tools to promote discussion allowing us to explore these questions and compare stakeholder descriptions of the intervention with descriptions in the literature. Following good practice in community-based participatory research^29^ ^30^ we adopted the stance of researchers as partners in a process of reflection and learning, making it explicit that we needed to gain a better understanding of what actually happens during a support intervention by listening to people who provide it.

Participants helped us to record information using a variety of different formats. We made notes, used flip charts, audio recorded some discussions, and used post-it notes to co-produce themes. The events were enjoyable and feedback from participants was positive, suggesting they felt valued and affirmed in their role. This was evidenced by ongoing participation from the same individuals and organisations at subsequent events, as well as invitations for longer term collaboration on funding proposals and programme evaluations.

Themes relating to peer support were collaboratively and iteratively developed. Information from the first event was compiled by members of an umbrella Consortium representing most of the voluntary agencies in the local area, working alongside members of the review team. The Consortium disseminated the preliminary information to participating organisations for feedback. Information in each theme was discussed at subsequent Advisory Network events to produce a comprehensive description of the intervention including the peer-support environment, the characteristics of the implementers, and the implementation process. The discussion moved from description of the complex intervention of peer support, to active questioning of what makes it work. At each stage, we also played ‘devil's advocate’ by asking people to tell stories of the challenges of providing support, ranging from concerns about individual capability to issues in dealing with systems. The network worked with a local artist to capture the process of peer support which was more widely disseminated to workers and clients in community settings for participant validation.

**Figure 1:**
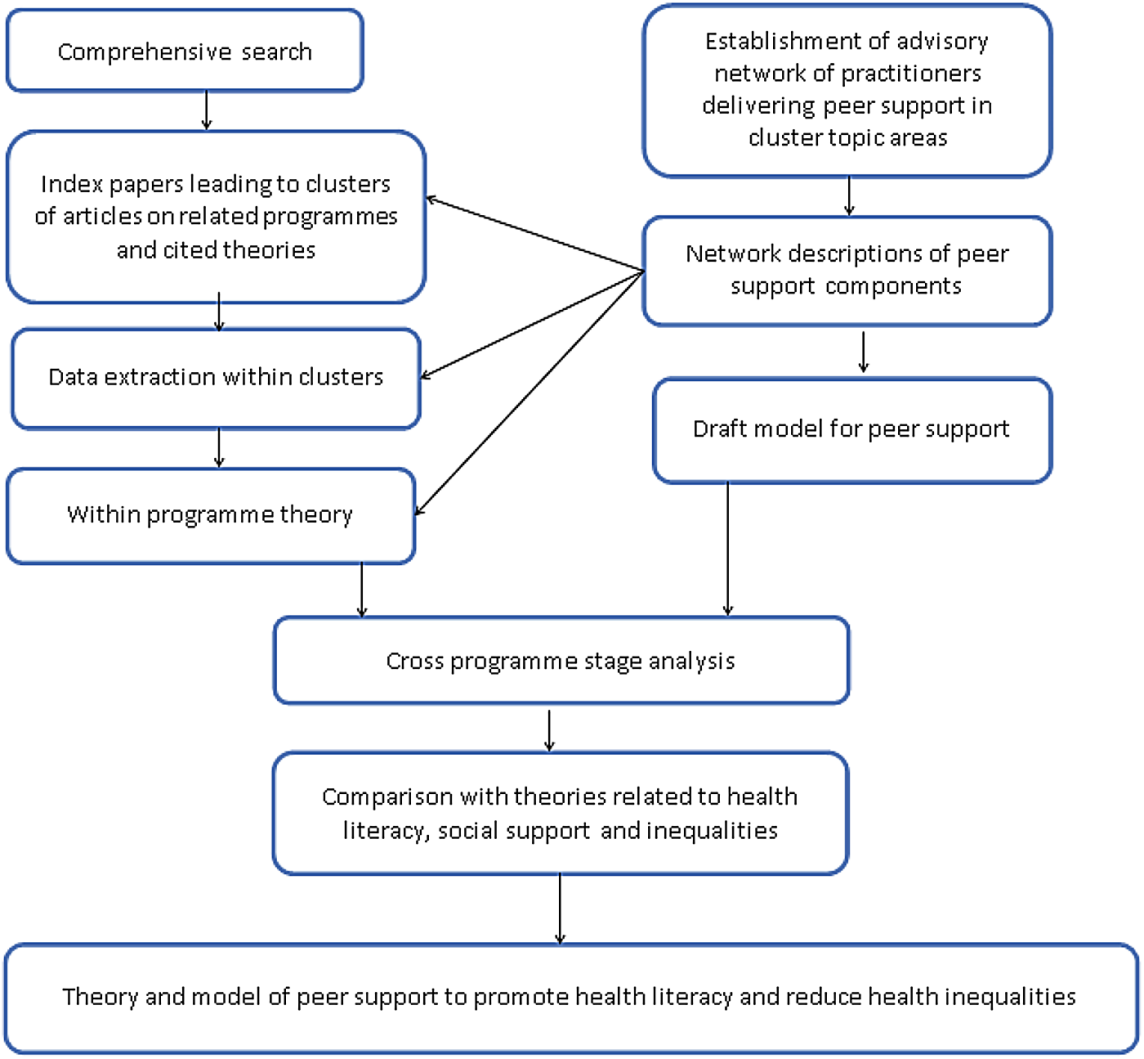
Stages to participatory realist synthesis.

The model produced by the Advisory Network described the components of a successful intervention and the theory of change for peer-support interventions. The model was used to guide the identification of relevant data from published studies. In many cases however, a single publication did not adequately report on all aspects of the intervention or programme. We therefore used a variety of search techniques to identify papers or other research outputs that related to a single study.^28^ For each health topic, we constructed a ‘cluster’ of data that included an index paper (key pearl citation) which was linked, through supplementary searches, to at least two or more additional papers from the same study. Theories that were explicitly used and/or cited within studies in each cluster were noted. Network descriptions of the complex intervention were compared with the literature within each topic cluster, to identify how, why and when interventions worked in different settings with different populations. We went on to develop propositions stating how the surrounding context, population and timing influenced the trajectory of the intervention. This phase was followed by cross-cluster analysis to determine if the emerging programme theory for peer support was represented across different health topics. We organised the data in relation to the stages of developing and implementing the interventions. Data from the clusters were used to construct a definition of each programme stage. Context–mechanism–outcome (CMO) chains from all clusters were used to populate the programme stages and the propositions were refined each programme stage (figure 1). The final product was a guide showing the processes needed at each stage of design and implementation to support effective interventions across different contexts, as well as the necessary components for peer-support interventions.

## Results—how participation added to the review

Participation throughout the review helped us to (1) identify active components of the intervention; (2) describe feedback loops where interactions influenced success of the intervention; (3) identify instances of tailoring the intervention in the literature; (4) identify proximal outcomes and (5) analyse the ways in which context affects the intervention at different levels. The network also played a key role in the development of theory. How participants contributed to each of these aspects will be briefly described.

### Finding active components for complex interventions

The network descriptions of peer-support components matched the comprehensive conceptual framework developed by Dennis,^31^ with one notable exception. Dennis listed four types of peer support found in social science research: emotional support, affirmational support, informational support and instrumental support. She found that in a healthcare context, the first three are extensively researched, while ‘the literature clearly demonstrated that that peer support primarily occurs without the provision of instrumental support’ (ref. 31, p.325). In contrast, there was consensus across the Advisory Network that a key attribute for successful peer support is instrumental support—the giving of practical help and tangible aid. Further, where the health literature privileged information giving as the primary mode of support, the Advisory Network emphasised that information is not effective unless it is preceded by the establishment of a relationship which is characterised by trust in the sender of the health messages. Peer supporters emphasised that establishing relationships and trust was key to effective support, saying that they often engaged with individuals on issues that were unrelated to health in the first instance. This allowed the individual to feel in control of discussing what was most important, signalling that they were involved in a relationship of parity with the peer-support worker. As participants were given the lead in identifying issues, the provision of health information in many cases did not occur until patients or clients were ready for it. This type of opportunistic information-giving was not acknowledged as an important component of peer-support interventions in quantitative studies.

We used the network perspective to return to the literature. We found that practical support was identified as important across multiple studies, but not evaluated. Some of our included studies also acknowledged that giving ‘scientific’ and technical information was ineffective without practical help and modelling.^32–35^

### Describing feedback loops

The Network described a feedback loop where going places with people and doing things with them enabled the development of social connections and self-help skills, which in turn triggered the mechanism of increased confidence. Practical support, provided initially by the peer-support worker and subsequently supplemented by expanding social networks, enabled the development of skills for interacting with systems to address health needs. Each successful encounter further increased confidence and motivation to continue to develop capabilities. Feedback loops, however, only occurred when there were established mechanisms of trust and supportive relationships.

In the literature, trust was acknowledged in some qualitative research articles to be a potentially influencing mechanism. The controlled trials, in contrast, focused on peers having similar characteristics but did not assess whether being of the same ethnicity, age, gender or speaking the same language engendered trust.^36^ The importance of monitoring the process of establishing and maintaining relationships was described extensively in some of the qualitative research^34^ but was not operationalised in the quantitative studies. This may be one of many factors that account for the variations in the effectiveness of peer support that were identified in a recent review of community engagement.^37^

### The importance of tailoring interventions

The Advisory Network agreed that tailoring peer support should be considered a standard approach to implementation. This prompted us to review the degree of tailoring in the published studies. In the trials, the degree of tailoring was not considered important. Qualitative studies, however, noted that expectations to provide a standard package of support frustrated workers.^38^ ^39^ Further discussion with the Network indicated that while tailoring of interventions was considered to be a good practice, it was not always reported to funders. This has implications in terms of the amount of tailoring that is reported in effectiveness studies and subsequently available for analysis in reviews.

### Identifying proximal outcomes

Researchers and practitioners in the network had different definitions of valued outcomes. A raft of process outcomes were agreed across the Network which were often social in nature. For example, support to develop or expand social networks was related to increased confidence in ability to tackle health issues; using social networks to construct meaning from health information enabled people to develop critical health literacy; and developing trust enabled people to share issues about life circumstances, which in turn enabled them to feel supported in addressing health. While the Network stated that these social outcomes are a precondition for achieving health and well-being, these were not evaluated in the trials. Studies using quantitative designs used longer term health outcomes, skipping over the proximal social outcomes, and most included studies evaluated impact within 2 years or less after implementation. In systematic reviews, there is a risk that interventions will be judged ineffective using inappropriately longer term criteria, when they may actually be effective if measured against shorter term proximal social outcomes. A recent systematic review has noted the disconnect between evaluations of social process and health research, calling for the inclusion of social dimensions in health evaluations.^40^

### How context affects implementation at different levels

Initially, nearly all of our included studies appeared to be assessing community-based peer support at the point of implementation, evaluating the relative success of the peer-support worker as the vehicle for delivering the intervention. The Advisory Network, however, emphasised the importance of allowing peer-support workers to exercise autonomy and judgement. This spurred us to return to the literature and look for the broader context, in terms of organisational attitudes towards workers, organisation support systems, and degree of autonomy given to them. A pattern emerged from the literature, which we eventually characterised as an ‘equity context’. In brief, settings where organisations value the experiential knowledge of the worker and their community were more likely to produce successful interventions than those where organisations controlled the message and the mode of delivery. Organisational control was described in two of our included reviews^35^ ^39^ but the development of the concept of an equity context was facilitated by our participants.

## Discussion

Our approach to stakeholder involvement revealed that lack of involvement in primary studies, as well as in systematic reviews, may compromise the validity of reviews in several ways. First, our comparison of user-generated components with previous research illustrated that researchers may not identify all of the important components. Second, the type and quality of interactions between providers and beneficiaries may be hugely influential, but not always considered when reviewing treatment fidelity. Third, short-term outcomes can mediate or moderate impact, but they may be ignored, reducing the explanatory power of the review. And finally, variation in the surrounding context in terms of organisational and social support for the intervention may have a greater influence than normally acknowledged in effectiveness reviews. We also found that in realist reviews, it is worth looking at the conceptual security of the key concepts, to determine whether there is a shared understanding of the phenomenon. In effectiveness reviews of complex interventions, it may also be critical to go beyond the definitions of the intervention that are normally derived from published studies, and question whether all important components of the intervention are operationalised from the perspective of different stakeholders.

Although we found that sustained and equal involvement in the review process substantially contributed to review quality, it has to be asked whether this level of participation should be routinely included in reviews of complex interventions. There are issues of time and cost, as well as researcher skills in participatory working; and the circumstances in which the review is commissioned.

Does participation really take more time and cost more? This is heavily dependent on the prior relationship with reviewers, as well as the skill set in the review team and the review topic.^41^ In our review team, one member had prior experience of participatory evaluation with some network members; and three people on the team had experience of PPI. Further, the Consortium members who were commissioned to help us build the network had excellent relationships with providers, peer supporters and clients and this credibility and trust was essential in terms of ‘sponsoring’ our project with people who were unknown to the academic team. Members of the team were also experienced in participatory working. Participatory approaches to research require a distinct set of skills^41^ ^42^ and we would caution review teams against assuming that ‘anyone can do participation’ as use of the approach without the skills set can do harm in terms of raising expectations without subsequently including participant views in the review.

Involving patients and the public in systematic review potentially suggests a broadening of what we might consider to be ‘expertise’, incorporating the experiential expertise of patients and carers into activities traditionally undertaken by ‘certified’ experts (by virtue of educational qualification). Yet how we define ‘expertise’ and who is classed as an expert is open to debate as knowledge and expertise are politically, socially and culturally influenced. As Jasanoff^43^ explains:

What operates as credible expertise in any society corresponds to its distinctive civic epistemology: the criteria by which members of that society systematically evaluate the validity of public knowledge. (ref. 43, p.394)

There are a growing number of examples of the changing dynamics between certified and experiential forms of knowledge, with expertise viewed as increasingly contested within late modern societies, often attributed to a democratisation of knowledge.^44–46^ In the health and sociological literature the emergence of the ‘lay expert’ (while an oxymoron) or expert patient indicates that the experiential expertise of patients and carers is increasingly valued in health research.^47^ Despite this, it has been argued that experiential expertise might be regarded as subjective opinion and written off as inferior or ‘misguided ways of knowing’.^48^ As such, the legitimacy of experiential expertise in healthcare and social care research has been queried. However, current research on integrated knowledge translation shows that knowledge production in any form is not solely the product of scientific expertise but a complex process of knowledge co-creation and the inherent value of practical knowledge in generating appropriate interventions for particular contexts.^41^ ^49^ The perceived importance of the review topic, and the motivations of the review commissioners, is also key to involving patients and the public. Our topic—community engagement and peer support—has been the focus of recent national policy, as well as has raised concerns across community organisations that provide the service. With the recent move of public health to local authorities in the UK, and the recognition of the high cost of long-term and chronic conditions, there has been a drive to engage community organisations in promoting self-management.^50^ As a result, our participating organisations were highly motivated to provide information on what works and what doesn't work, as they understand the need to compile an evidence base.

Even with experienced practitioners and timely topics, there are several challenges with participatory reviewing, which include the role of the reviewer, the timing of involvement, and facilitating participation in ‘academic’ review. In participatory reviewing, the role of the researcher changes from being that of the expert to handing control over to the participants. This occurs because a successful participatory review promotes deliberative dialogue.^51^ Deliberative methods involve consumers in carefully weighing the propositions for what works and promote exchange of different points of view in order to arrive at a decision. This sort of dialogue erases the role of privileged academics.^52^ Concerns about preserving the integrity of science can challenge funding bodies when they try to involve providers and service users in the ‘front end’ of science.^53^ There tends to be an epistemological clash between empirical approaches to enquiry and postpositivist approaches. Striking a balance between empirical evidence and social systems and subjective values can only be achieved through reflexivity—which in this context means the ability to be aware of the types of knowledge that are being privileged at different stages of the review and being able to justify the reasons for gravitating toward different forms of knowledge during analysis.

In terms of the timing of involvement, our network was not involved in developing the funding proposal for the review, which is far from ideal. Neglecting involvement during proposal development risks a loss of ownership in the topic and creating the academic review team before bringing others on board can create an ‘us and them’ situation. This leads to involvement on the level of consultation, where opinions are occasionally sought but not always used.^54^ Prior relationships with participants and informal discussion about the proposal compensated for lack of early involvement, but we would normally involve participants at the first stage of prioritising the topic, problem formulation and proposal development.

## Conclusions

Peer support has been characterised as an intervention in search of a theory,^55^ while conceptual models for health literacy are relatively new^56^ and the relationship between peer support and health inequality is entirely new territory. Sustained participation with Network members who co-produced descriptions of the complex intervention and programme theory enabled us to conduct a more in-depth conceptual analysis than solely relying on the literature.

In considering information from the Advisory Network and from the published evidence we found a clear difference in emphasis. The published literature characterised peer-support interventions as brief and episodic, emphasising delivery of a health message by someone who is perceived to have similar characteristics. In contrast, the network emphasised that the process of peer support begins with a worker but is more often successful when the worker enables the beneficiary to make connections over time and supports them in being in control of the process.

Sustained involvement of an Advisory Network produced information on previously unacknowledged and important components of the intervention. The interaction enabled the academic team identify these components in published studies, understand how they worked together to produce proximal and more distal outcomes, and recognise that the achievement of short-term social outcomes was in many cases a precondition for tackling more challenging health issues. The theoretical model produced by the network was instrumental in developing the final theory to explain what was necessary for successful peer support.

We originally envisaged that our community experts would be involved in the realist review in tandem with the review team, with community members describing the key components of culturally supported interventions, while the academic team identified empirically supported interventions from the literature. Our assumption was that both sources of information would contribute equally to the development of a peer-support model. However we found that published accounts gave very little description or detail of the interventions and it was often difficult to identify what had been done, what the peer support actually consisted of and what were the components of the intervention. As a result data from the Advisory Network were instrumental in helping the team to address incompleteness of reporting in the published studies.

In summary, participatory reviewing can add value to a systematic review when:

- The researchers are skilled in including a wider range of stakeholders and equally valuing different sources of knowledge;
- The components of the intervention or programme need to be clarified;
- The underlying theory for the programme needs to be better articulated;
- There are questions about whether trials have captured all of the valued outcomes;
- More insight is needed on the relationship between intervention and outcome;
- There are questions as to whether the interventions are culturally acceptable and appropriate.

Without the input from our network, we would have relied more heavily on published conceptual reviews, which were shown to be missing some of the key components of the intervention. The participation and level of involvement has therefore contributed to strengthening the validity of the theory, potentially increasing the utility and transferability of the review. Participatory reviewing can for some topics and situations produce a more contextually valid synthesis.

**What is already known on this subject**

- Patient and public involvement (PPI) has the potential to make systematic reviews more relevant, accessible, accountable, and acceptable to end users but little is known about how they can contribute to reviews of complex interventions.
- Involvement of stakeholders in reviews is intermittent, usually occurring at the beginning and end of the process.
- The evidence base for the utility of PPI, in terms of its’ potential to improve reviews, remains weak.

**What this study adds**

- Sustained involvement throughout a review can assist in the identification of components of complex interventions, enabling conceptual analysis particularly where original reporting was thin.
- Using participatory approaches to realist review can maximise engagement in building explanations for variation in how, when, why and where complex interventions work.
